# A Profile of Orthopedic Education in Emergency Medicine Residency Programs in the United States

**DOI:** 10.7759/cureus.49257

**Published:** 2023-11-22

**Authors:** Elizabeth B Werley, Krystin Miller, David P Way, Christina Hajicharalambous, Carmen J Martinez Martinez, Caroline Molins

**Affiliations:** 1 Department of Emergency Medicine, Penn State College of Medicine, Hershey, USA; 2 Department of Emergency Medicine, The Ohio State University College of Medicine, Columbus, USA; 3 Department of Emergency Trauma, Hackensack Meridian Health (HMH) Hackensack University Medical Center, Hackensack, USA; 4 Department of Emergency Medicine, University of South Alabama College of Medicine, Mobile, USA

**Keywords:** emergency medicine, medical education, curriculum, musculoskeletal, orthopedics

## Abstract

Introduction

Emergency medicine (EM) residents throughout the United States are required to become skilled at performing a robust list of select orthopedic procedures, as well as learn how to diagnose and manage patients with musculoskeletal complaints. However, EM residency programs vary significantly in how they teach orthopedics and the content they cover. The purpose of this study was to profile the orthopedic education received by emergency medicine residents in United States residency programs.

Methods

We developed a survey based on accreditation requirements and The Model of the Clinical Practice of Emergency Medicine. The survey was designed to gather detailed information about the orthopedic education provided to EM residents. The survey was sent to EM program directors or their designees at all 263 accredited EM residency programs across the United States between October 2020 to January 2021.

Results

We attained a 34.6% (91 of 260) adjusted response rate with adequate representation of relevant program characteristics such as region, accreditation status, program length, size, and setting. Most (63.7%) responding programs required an orthopedics rotation during the intern year. These required orthopedic rotations were primarily four weeks in duration. The most common methods for teaching orthopedic topics included didactics (97.8%), procedures on live patients under supervision (73.3%), and assigned reading materials in textbooks or manuals (68.9%).

Conclusion

The orthopedic education received by EM residents in the United States is strikingly variable, with residency programs having to develop custom curricula to teach orthopedics content based on the resources available to them. Future efforts should be directed toward creating a universal curriculum that addresses accreditation and EM practice standards.

## Introduction

In the United States, musculoskeletal complaints and orthopedic injuries account for 17.8 million, or nearly 14%, of all emergency department (ED) visits each year, making it one of the most common presentations to the ED [1]. Significant morbidity and disability can result from inappropriately managed musculoskeletal injuries. Two of the top 10 diagnoses involved in ED malpractice claims involve fractures of the radius or ulna and fractures of the tibia or fibula [2]. For these reasons, it is imperative that emergency medicine (EM) physicians learn and maintain proficiency in musculoskeletal medicine and the management of orthopedic injuries, particularly fractures and dislocations.

Education of the musculoskeletal system and its associated pathology is relevant to numerous medical specialties yet is quite variable throughout United States medical school curricula [3-4]. Subsequently, most training in the management of orthopedic injuries begins in residency [4]. The current Accreditation Council for Graduate Medical Education (ACGME) Residency Review Committee (RRC) for EM highlights the importance of orthopedic (ortho) education by identifying dislocation reduction as a key index procedure. However, RRC requirements remain somewhat vague in that they only require completing 10 unspecified dislocation reductions by graduation [5]. The American Board of Emergency Medicine’s (ABEM's) 2019 Model of the Clinical Practice of Emergency Medicine also provides guidance for ortho education in that it includes management of musculoskeletal complaints, orthopedic injuries, and fracture and dislocation reductions as part of the core content of EM [6].

Despite the prevalence of orthopedic injuries seen clinically and the focus on orthopedic pathology by the ACGME and ABEM, orthopedic education continues to be an area of concern for EM [7]. Many graduates feel unprepared to manage fractures in the ED upon residency graduation [7-8]. Prior studies have proposed various methods for incorporating orthopedic content into the EM curriculum, including but not limited to the provision of clinical rotations dedicated to caring for patients with ortho injuries or musculoskeletal complaints. Although some researchers have attempted to shed light on the effectiveness of clinical orthopedic supplements in EM education, there is limited knowledge of precisely how orthopedics is taught in EM [9-15]. The purpose of this study was to profile how ortho education is provided in EM residency programs across the United States. Our hypothesis was that we would observe considerable heterogeneity between programs beyond the set ACGME standards related to orthopedic/musculoskeletal education.

## Materials and methods

This was a cross-sectional survey-based study performed from October 2020 to January 2021. This research was determined to be exempt from human subjects review by the Institutional Review Board at Penn State College of Medicine (Study ID: STUDY00013576).

Population and sampling

Our target population was individuals from EM residency programs who were responsible for the orthopedic education of their residents. For most, but not all programs, this was the program director (PD). We identified 263 ACGME-accredited EM residency programs established before January 2020 using the ACGME public website “List of Programs by Specialty” search function and the American Medical Association's (AMA's) Fellowship and Residency Electronic Interactive Database (FREIDA) [16-17]. Using these resources along with the Council of Residency Directors in Emergency Medicine (CORD-EM) directory, the Emergency Medicine Residents’ Association (EMRA) residency search function, EMRA Match, and program websites, we assembled a roster of PDs and their contact information [18-19]. PDs were contacted by email with an overview of the study and were provided an opportunity to designate an alternative faculty member to participate on the program's behalf (i.e., another faculty member who oversees orthopedic/musculoskeletal curricular content).

Instrument development

The authors (five EM faculty physicians and a survey methodologist) developed a survey instrument in an iterative fashion. The authors based the instrument primarily upon three sources: ACGME requirements for EM, ortho/musculoskeletal content from the ABEM Model of the Clinical Practice of Emergency Medicine, and a review of other existing literature [5-6,7-8,13]. The resulting instrument was designed to gather detailed information about the orthopedic education provided to EM residents. We asked respondents how much time was allocated to orthopedic education, the methods used to teach fracture and dislocation management, the nature of any ortho rotations (required or elective), whether the program had access to an ortho residency program or department, and the teaching methods used to teach about ortho/musculoskeletal complaints. Finally, we asked respondents to rate their graduating residents’ competencies in performing reduction and splinting procedures.

The survey was reviewed and edited among four EM PDs, or their designees, at each of the authors’ home institutions before distribution to the broader population. These PDs also participated in the final study. Three changes were made on the basis of PD feedback: 1) We dropped items related to the number of fracture and dislocation reductions reported to the ACGME since these were considered too difficult to report without a significant investment of respondent time; 2) We added methods of instruction and settings (rotations) in which ortho is taught; and 3) We revised our response options for items related to PDs ratings of graduating resident performance from milestones to competencies (see Appendix 1).

To profile program types, we selected vital program demographics from the websites that characterize programs, including region of the country (as defined by the Association of American Medical Colleges (AAMC)), accreditation status (initial or continuing accreditation), program length (three or four years), and program setting as defined by the AMA-FREIDA website (university-based, university-affiliated, community-based or military) [17,20]. We also used the total number of residents to characterize a program’s size [17]. Using meaningful gaps in the distribution of program size, we defined small programs as <33 residents with an average of 25 (SD=5.0), mid-size programs as having between 34 and 45 residents with an average of 39 (SD=3.5), and large programs as having >46 residents with an average of 58 (SD=10.8) [16].

Survey implementation

We used Dillman’s Tailored Design Method to guide the implementation of our survey [21]. This included advanced notification of the study and its purpose, along with reminders spaced at two-week intervals for a period of two months from October through December 2020. To boost our rate of return, we also posted survey links on the CORD-EM PDs listserv [22] with a request embedded in the survey to self-identify the program they represent. Finally, in January 2021, the research team divided up the list of non-respondents, and members of the team sent personal emails to individual PDs on their list with a request to complete the linked survey. These individuals were also asked to self-identify the person completing the survey. We used the Qualtrics electronic survey platform (Qualtrics, Provo, United States) to disseminate our survey.

Data management and analyses

Descriptive statistics were used to profile various types of EM programs. Inferential statistics were used for comparing program profiles. All analyses were performed with IBM SPSS Statistics for Windows, Version 27 (Released 2020; IBM Corp., Armonk, New York, United States). Duplicate responses (from the same program) were consolidated or eliminated. We used Chi Square tests of association as a method of checking for response bias by comparing respondents to non-respondents across the key program demographics [23].

We sorted programs into groups based on whether they required residents to complete an ortho rotation and/or whether they offered ortho to their residents as elective rotations. This resulted in four groups: 1) programs that require an ortho rotation and offer electives, 2) programs that require an ortho rotation but do not offer electives, 3) programs that do not have an ortho requirement but offer electives, and 4) programs that neither require an ortho rotation nor offer electives. These groups were compared on both program characteristics and outcomes. For comparing outcomes, we used a structured analysis and Bonferroni correction to preserve power and control for type 1 errors. The structured analysis involved first running global comparisons with one-way analysis of variance (ANOVAs) comparing mean scores of the aggregated upper and lower extremity competency ratings. If these were statistically significant at the p<0.025 level, we conducted individual one-way ANOVAs on the individual competence ratings of joint dislocations/reductions and fracture reductions. Student Newman-Keuls post hoc tests were then applied to explain differences between groups observed on individual competency items.

## Results

We received 110 survey responses, of which 27 were incomplete, and 12 were duplicates. We also received three responses for which the associated program was unidentifiable. Where possible, incomplete and/or duplicate responses were consolidated into one response. Unidentified responses and those missing more than 50% of answered questions were eliminated, leaving us with 91 complete survey responses and an adjusted return rate of 91 out of 263 programs, or 34.6%. The demographic profile of programs described by our respondents were primarily three years in length (78%), university-based (46%), from the northeast region (35%), small in size, with an average of 25 residents (37%) and had achieved continued-accreditation (80%).

Bias analysis and program characteristics

Table 1 shows the EM program profile of 91 programs that responded to our survey about ortho education compared to non-respondents on five key program characteristics. Chi-Square Tests of Association suggested that respondents to our survey were representative of the larger population with regard to accreditation status and program length. However, our sample was not representative of the population with regard to region, program setting, and program size. We received slightly more responses from programs located in the northeast region of the country (35%, 32 of 91) and slightly fewer from the western region (13.2%, 12 of 92). However, the percentage of responses from each region (of the total available) was relatively similar from all four regions of the US. Since we did not receive any responses from military programs, in order to conduct this bias analysis, we incorporated military programs with community-based programs (adjusted community-based). These results suggested that our respondents were more representative of university-based programs and that university-affiliated and community-based programs (including military) were slightly under-represented. Our sample was also not representative when it came to program size, suggesting that we received slightly more responses than expected from large programs and fewer from small programs. In both cases of detected response bias, the associated effect sizes (Cramer’s Phi) were considered small (Table 1) [24].

**Table 1:** Program profile of 91 programs who responded to our survey about orthopedic education compared to non-respondents on five program characteristics: (a) region of the US, (b) accreditation status, (c) program length, (d) program setting, and (e) program size. Population data represents the 263 programs that were surveyed *Military programs were re-coded to community-based programs (adjusted community-based) for this analysis since we received no responses from these programs. CV = Cramer’s V (or Phi) is a measure of the associated effect sizes for the chi-square test of association in which a small effect is defined as 0.10, a medium effect as 0.30, and a large effect as 0.50 [24].

		Respondents		Nonrespondents		Population	
(a) Region		N	%	N	%	N	%
	Northeast	32	41	46	59	78	29.7
	South	24	29.3	58	70.7	82	31.2
	Central	23	34.3	44	65.7	67	25.5
	West	12	33.3	24	66.7	36	13.7
	Total	91	34.6	172	65.4	263	100
	X^2^ =1.19, df=3, p=0.76, CV=0.058						
(b) Accreditation status		N	%	N	%	N	%
	Continued	71	35.9	127	64.1	198	75.3
	Initial	20	30.8	45	69.2	65	24.7
	Total	91	34.6	172	65.4	263	100
	X^2^ =0.15, df=1, p=0.70, CV=0.028						
(c) Program length		N	%	N	%	N	%
	3-year	73	35.8	131	64.2	204	77.6
	4-year	18	30.5	41	69.5	59	22.4
	Total	91	34.6	172	65.4	263	100
	X^2 ^=0.15, df=1, p=0.70, CV=0.028						
(d) Program setting*		N	%	N	%	N	%
	Community-based	13	29.5	31	70.5	44	16.7
	Military	0	0	5	100	5	1.9
	Adjusted community-based	13	26.5	36	73.5	49	18.6
	University-affiliated	36	30.3	83	69.7	119	45.2
	University-based	42	44.2	53	55.8	95	36.1
	Total	91	34.6	172	65.4	263	100
	X^2 ^=2.99, df=2, p=0.22, CV=0.092						
(e) Program size		N	%	N	%	N	%
	Small	34	26.2	96	73.8	130	49.4
	Mid-size	31	39.2	48	60.8	79	30
	Large	26	48.1	28	51.9	54	20.5
	Total	91	34.6	172	65.4	263	100
	X^2^ =4.41, df=2, p=0.11; CV=0.111						

To determine the availability of orthopedic physicians to contribute to EM resident education, we asked respondents if their institution included an ortho residency program and, if not, did their ortho department hosted residents from outside their department. Most respondents (61 of 91, 67%) told us that their institution included an ortho residency program. An additional 11 of 91 (12%) said that their institution did not have an ortho residency, but their ortho department served as a rotation site for other residency programs (including EM residents). The remaining 19 of 91 (21%) had no ortho residency, nor did they have an ortho department that hosted non-ortho residents. To simplify subsequent analyses, we combined those with access to either an ortho residency or department and thought of them collectively as EM residency programs with access to orthopedic-trained specialists as a potential resource to supplement the program’s ortho education.

Setting of orthopedic rotations

**Figure 1:**
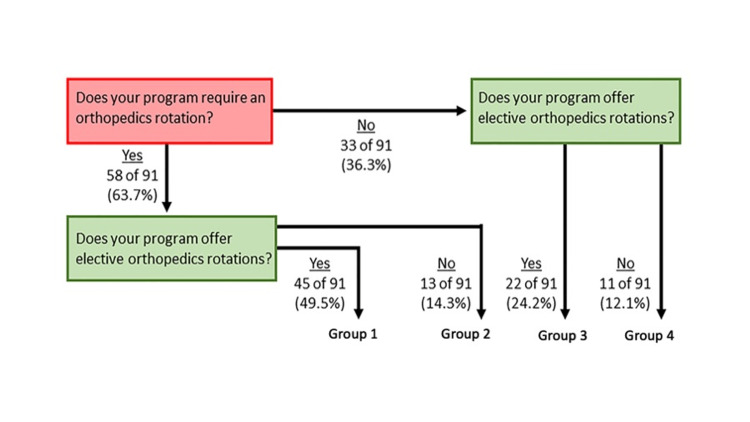
Number of programs that offer required orthopedic rotation vs elective rotation

Orthopedic/musculoskeletal content is covered in a variety of settings in EM: the classroom, simulation laboratory, clinical settings, and through self-directed learning. In the clinical setting, orthopedic education may exist in the form of a program-required rotation or elective rotation. Figure 1 is a flow diagram that describes the number of programs that require an orthopedics rotation and/or offer elective orthopedics rotations.

Table 2 shows the frequencies and percentages (in parentheses) of rotation types, settings, and whether they are dedicated or not for programs with various configurations of required and/or elective orthopedic rotation experiences for EM residents.

**Table 2:** Frequencies and percentages (in parentheses) of rotation types, settings, and whether they are dedicated or not for programs with various configurations of required and/or elective orthopedic rotation experiences for EM residents *Group 3 ratings are significantly lower than the other three groups (p<0.01) Mean (Mn) and standard deviation (SD) are shown for time on clinical rotations and average competency ratings. EM: Emergency medicine

	Group 1		Group 2	Group 3	Group 4
Service type(s)	Required	Electives	Required	Electives	Neither
Consult service	32 (23.4)	24 (19.4)	6 (22.2)	12 (19.7)	
Emergency department	35 (25.5)	23 (18.5)	7 (25.9)	9 (14.8)	11 (100)
Inpatient service	18 (13.1)	6 (4.8)	6 (22.2)	8 (13.1)	
Operating room	9 (6.6)	5 (4.0)	1 (3.7)	2 (3.3)	
Orthopedic clinic	23 (16.8)	18 (16.1)	4 (14.8)	10 (16.4)	
Pediatric ortho service	5 (3.6)	6 (4.8)		3 (4.9)	
Sports medicine	14 (10.2)	28 (22.6)	3 (11.1)	11 (18.0)	
Urgent care	1 (0.7)	2 (1.6)			
Sports teams or event care		12 (9.7)		6 (9.8)	
*TOTAL*	137 (100)	124 (100)	27 (100)	61 (100)	11 (100)
Rotation setting(s)					
Academic health center	35 (52.2)	31 (52.5)	6 (42.9)	13 (56.5)	
Community facility	23 (34.3)	21 (35.6)	7 (50.0)	6 (26.1)	
County facility	9 (13.4)	7 (11.9)	1 (7.1)	2 (8.7)	
Military or VA					
Missing				2 (8.7)	
*TOTAL*	67 (100)	59 (100)	14 (100)	23 (100)	11 (100)
Access to ortho residency/department	41 (91.1)		9 (69.2)	16 (72.7)	6 (54.5)
Rotations involving dedicated ortho shifts					
Required only	6 (13.3)		6 (46.2)		
Elective only		2 (0.4)		12 (54.5)	
Both	30 (66.7)				
No	7 (15.6)		7 (53.8)	10 (45.5)	
*TOTAL*	45 (100)		13 (100)	22 (100)	11 (100)
Time on clinical rotations	Mn (SD)		Mn (SD)	Mn (SD)	Mn (SD)
Average weeks-required	3.47 (1.35)		2.92 (1.12)	0.00	0.00
Average weeks-elective		5.29 (2.82)	0.00	4.95 (3.12)	0.00
Total average clinical weeks	8.38 (3.42)		2.92 (1.12)	4.95 (3.12)	0.00
Average competency ratings	N=42		N=11	N=15	N=7
Upper extremity composite	3.23 (0.62)		3.36 (0.36)	2.80 (0.64)*	3.64 (0.43)
Lower extremity composite	3.24 (0.59)		3.30 (0.56)	2.63 (0.76)*	3.61 (0.45)

The location of both required and elective rotations varies by program. In some programs, residents learned ortho skills through rotations on ortho units: departments, clinics, consult services, operating rooms, or inpatient services. Other programs provided ortho education through resident rotations in the ED. Among those programs, some relied on conventional EM rotations and chance resident encounters involving patients with orthopedic or musculoskeletal injuries. Still, others provided dedicated ortho shifts on which the resident took primary responsibility for patients with orthopedic injuries or musculoskeletal-related complaints without any other clinical responsibilities.

We found that those programs that had access to an ortho residency program or department were 86% more likely to require an ortho rotation (LR 1.65, 95% CI (0.78,0.92)) and were 85% more likely to offer ortho rotations as electives (LR 1.50, 95% CI (0.79,0.90)) [25]. Nearly two-thirds of respondents (58 of 91, 63.7%) required their residents to complete an ortho rotation. Of those 58 programs that required it, 42 of 58 (72.4%) conducted this required rotation in the ED itself. Alternatively, required ortho rotations were also conducted on orthopedic-based consult services (38 of 58, 55%) or the ortho clinic (27 of 58, 46.6%). Nearly three-fourths of respondents (67 of 91, 73.6%) offered elective rotations to their residents. Elective rotations were offered in a variety of locations but primarily took place in sports medicine clinics (39 of 67, 58.2%), orthopedic consult services (36 of 67, 53.7%), the ED (32 of 67, 47.8%), or the orthopedics clinic (28 of 67, 41.8%). For those electives that took place in the ED, all but six of them said that these were dedicated orthopedic rotations as opposed to chance encounters on shift.

Timing of orthopedic rotations

Among the 63.7% (56 of 91) of programs that required an ortho rotation, most (64%, 37 of 56) required that residents have that rotation during the first post-graduate year. However, when ortho rotations were offered as electives, they were more likely to occur in the second post-graduate year or later. As can be seen in Table 2, for programs that required ortho rotations, the average length of time for that rotation (Groups 1 and 2) ranged from 2.92 to 3.47 weeks. For programs that offered elective ortho rotations (Groups 1 and 3), the average length of time for that rotation ranged from 4.95 to 5.29 weeks.

Methods of orthopedic education

Nearly all programs reported teaching orthopedic content through didactics, either lecture-based or small-group discussions (98%) (Table 3).

**Table 3:** Frequencies and percentages (in parentheses) of methods for teaching orthopedic content described by programs, by program length, access to the orthopedics department, and program setting *Community-based; ǂUniversity-affiliated; #University-based Differences highlighted in the narrative appear in bold italics. FOAMed: free open-access medical education; ED: Emergency department

Method	Total N=91 (%)	Program length		Ortho department access		Program setting		
		3-Year N=71 (%)	4-Year N=20 (%)	Yes N=72 (%)	No N=19 (%)	*Comm-Based N=13 (%)	‡U-Affiliate N=36 (%)	#U-Based N=42 (%)
Didactics: Lecture or small group	89 (98)	69 (97)	20 (100)	72 (100)	17 (90)	12 (92)	35 (97)	42 (100)
Procedures on live patients under supervision	67 (74)	53 (75)	14 (70)	51 (71)	16 (84)	10 (77)	25 (69)	32 (76)
Assigned reading materials in a textbook or manual	63 (69)	49 (69)	14 (70)	49 (68)	14 (74)	9 (69)	28 (78)	26 (62)
Assigned online learning modules or FOAMed materials	42 (46)	33 (47)	9 (45)	30 (42)	12 (63)	5 (39)	21 (58)	16 (38)
Instructional videos	41 (45)	30 (42)	11 (55)	30 (42)	11 (58)	7 (54)	19 (54)	15 (36)
Oral cases	40 (44)	31 (44)	9 (45)	28 (39)	12 (63)	7 (54)	20 (56)	13 (31)
Simulation on task trainers or manikins	29 (32)	25 (35)	4 (20)	21 (29)	2 (11)	6 (46)	11 (31)	12 (29)
Simulation with high-fidelity simulators	16 (18)	13 (18)	3 (15)	11 (15)	5 (26)	3 (23)	7 (19)	6 (14)
Other, Please specify:	9 (10)	8 (11)	1 (5)	7 (10)	2 (11)	2 (15)	4 (11)	3 (7)
Board review-type questions	1 (1)	1 (1)			1 (5)	1 (8)		
ED-based ortho procedural rotation	1 (1)	1 (1)		1 (1)				1 (2)
Offered readings and websites	1 (1)	1 (1)		1 (1)				1 (2)
Procedural labs or workshops	5 (6)	4 (6)	1 (5)	4 (6)	1 (5)	1 (8)	3 (8)	1 (2)
Simulations on cadavers	1 (1)	1 (1)		1 (1)			1 (3)	

Besides didactics, other non-clinical teaching methods involved case presentations through oral cases (44%), demonstrations and practice on task-trainers/manikins (32%), or high-fidelity simulators (18%). A small number of programs reported the use of dedicated procedure labs or workshops (6%), while only one program used simulations on cadavers. Other common educational methods involved: assignments or expected use of self-directed learning materials such as textbooks or manuals (69%), online learning modules such as free open-access medical education (FOAMed) materials (46%), or instructional videos (45%). Nearly three-quarters of programs (74%) said that they teach orthopedic content through procedures performed on live patients under supervision. Most programs (81%) said the setting for this type of clinical teaching was through routine care of patients in the ED (data not shown).

Key differences between program types

With regard to teaching methods, we found a few key differences between programs based on program characteristics (Table 3). For instance, a greater percentage of three-year programs made use of simulations on task trainers or manikins than did four-year programs, while a larger proportion of four-year programs used instructional videos. Programs that lacked access to an orthopedic department tended to supplement clinical experiences with non-clinical teaching methods such as FOAMed materials, instructional videos, and oral cases. Finally, higher percentages of university-affiliated programs assigned either outside reading or FOAMed materials than did either university-based or community programs.

Program outcomes

Groups were significantly different in PDs’ mean competency ratings of performing reduction and/or splinting procedures required for both upper and lower extremities (adjusted p for significance was 0.025). These mean competency ratings were derived by taking the average ratings for joints in the upper and lower extremities separately. Subsequent analyses indicated that for the upper extremities, none of the competency items were significant on their own (adjusted p for significance is 0.05/5 = 0.01). For the lower extremities, subsequent analyses indicated that two items were significantly different: Knee/Patella and Foot/Digit (p=0.002 and 0.008, respectively). Post-hoc analyses revealed that group 3 (electives only) was rated significantly lower on orthopedic competency than the other three groups.

## Discussion

As hypothesized, we observed considerable heterogeneity in the manner in which orthopedics education is conducted by EM residency programs across the United States. Most importantly, we found that key differences in both the quantity and quality of orthopedics education were a function of the availability of resources, including the proximity of the EM program to an orthopedic residency program or clinical department. The programs with greater access to orthopedic resources were more likely to be university-based programs as opposed to university-affiliated or community-based programs. EM programs that had access to orthopedics residencies or departments were 86% more likely to require an orthopedics rotation of their residents and 85% more likely to offer orthopedics electives than those who did not have access. Furthermore, we observed that these programs were less likely to incorporate self-directed learning activities such as online learning modules or instructional videos.

While heterogeneity in ortho-education methods is not inherently a problem, when coupled with a lack of guidance in program standards, it creates heterogeneity in program outcomes. For instance, based on differences in ortho rotation offerings, we observed significant differences in how PDs rated the competency of their graduating residents in managing select orthopedic procedures. The guidance provided by the ACGME in the form of a minimum number of 10 unspecified dislocation reductions is insufficient for helping programs prepare their residents to manage the full spectrum of orthopedic complaints seen in the ED [5]. Content knowledge related to orthopedics can be found in several sections of ABEM’s Model of the Clinical Practice of Emergency Medicine, including the sections on non-traumatic musculoskeletal (11.0) and traumatic (18.0) disorders [6]. The ABEM model also provides a list of six orthopedic procedures that EM residents must become competent to perform in section 19.4.6.1-19.4.6.6 [6]. Without more standardization of educational expectations, programs will risk graduating residents who lack the ability to manage common orthopedic injuries.

While several attempts have been made to define content better or build standardized curricula for musculoskeletal/orthopedic topics, these efforts have not generalized well across programs since offerings of orthopedic content and training are contingent upon the unique set of resources available to a program or institution [9-11,14-15,25]. Chow et al. proposed a standard ortho curriculum that included goals, objectives, and educational strategies that could be adapted to any educational setting [25]. These proposed educational strategies involved textbooks, journal articles, online databases, FOAMed content (e.g., blogs, interactive clinical cases, podcasts, instructional videos), simulation-based sessions, and general didactic sessions; this could be used to supplement ortho education for programs that are not rich in ortho resources. We found that current EM residency programs made considerable use of the myriad types of educational strategies proposed by Chow; however, without more direct guidance in the form of curricular expectations, this type of standardized curriculum alone is not likely to ensure that EM residents attain these orthopedic competencies without corresponding clinical experience [25].

Standardizing clinical experiences for EM residents is a real challenge and may actually be impossible. We found that the primary driver of variability in an EM resident’s clinical ortho experience was entirely contingent upon the ortho resources available to the resident’s program. Clinical rotations and their structure varied widely based on whether an EM program had access to an ortho residency or an ortho department. Programs that had neither were often required to improvise by having residents participate in dedicated ortho shifts in the ED. Complicating standardization further are findings by others, such as Schmitz et al., who found key differences between the ortho education received by EM residents based on the time of day, components of the rotation, amount of resident autonomy, and patient volumes [9]. Another study that compared rotation structure by Briggs et al. found that both dedicated ortho rotations in the ED and primary care sports medicine rotations provided experiences comparable to rotations in an ortho department for EM residents [10]. Despite the potential for these alternative structures to be feasible options for EM residency programs to utilize for their trainees’ clinical ortho experiences, we believe that specific guidelines written in the form of both educational and clinical expectations are required to ensure that EM residents graduate with the competence to manage all orthopedic related complaints effectively.

Our study profiled the current curricular structure, teaching methods, and clinical exposure used for orthopedic education in EM residencies in the US. Future investigations should seek to determine whether the variety of orthopedic education structures and methods can be better standardized, ideally resulting in unified program requirements, broader than the current ACGME requirements, for graduating EM residents to improve their competence in musculoskeletal and orthopedic-related knowledge and skills.

Limitations

Despite our attempt at using the rigorous survey methods promoted by Dillman, our study yielded an adjusted return rate of 34.6% [21]. The bias analysis demonstrated that our response sample was representative of the larger population on four of six program demographics; however, our sample was skewed towards university-based and large programs. While we would have liked broader participation from PDs or their designees, we believe that the profile of EM orthopedic education provided by our response sample is a fair representation of how EM programs cover orthopedic education across all but the smallest community-based programs. Despite our desire for a greater rate of return, the 34.6% we achieved is considered respectable for a national survey study such as this one [26-28].

We distributed surveys and collected data during the COVID-19 pandemic. Since EM became the front line in fighting the pandemic, abrupt changes to educational content occurred across the country. To cover the influx of ill patients seen during COVID-19 and to accommodate those learners who became exposed and/or ill and had to quarantine or isolate, many programs resorted to canceling off-service and elective rotations, including orthopedic rotations [29-30]. Unfortunately, we were unable to fully discern whether participants’ responses reflected their pre-COVID orthopedics curriculum, or their adapted curriculum required by the pandemic, as some programs referenced both pre-COVID experiences and those that were occurring amid the pandemic.

## Conclusions

The profile of musculoskeletal/orthopedic education is quite varied across EM residency programs in the United States. Other than procedural numbers, there is no specific guidance from the ACGME on how to teach orthopedics effectively. The EM Model of the Clinical Practice of Emergency Medicine provides topics related to the types of patient encounters residents should see, but no other guidance directing programs on how to educate their learners on orthopedic/musculoskeletal content. There is no standard option; therefore, each program has been left to develop an individualized curriculum to cover this content, often within the constraints of local resources available to the program. While there are several similarities to the approach of EM programs on orthopedic procedures and educational content, perhaps more striking is the variability seen across this landscape and the potential that we are graduating residents without the competence to treat orthopedic injuries. We propose efforts by accrediting bodies to improve guidance to EM residency programs based on best practices and best evidence to include practical curriculum materials for those who lack access to orthopedic resources.

## Figures and Tables

**Figure 2:**
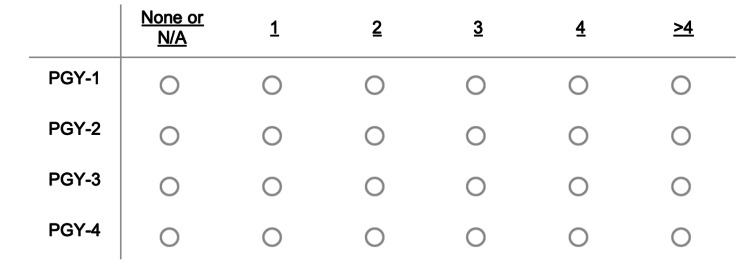
Answer choices

**Figure 3:**
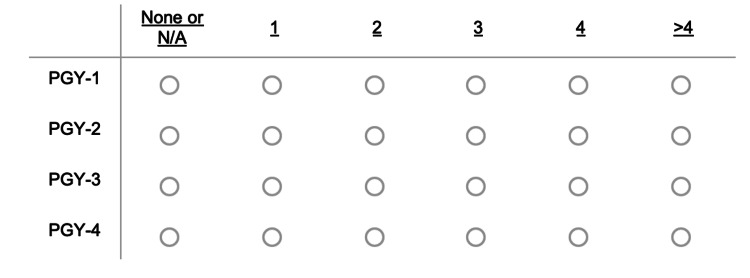
Answer Choices

**Figure 4:**
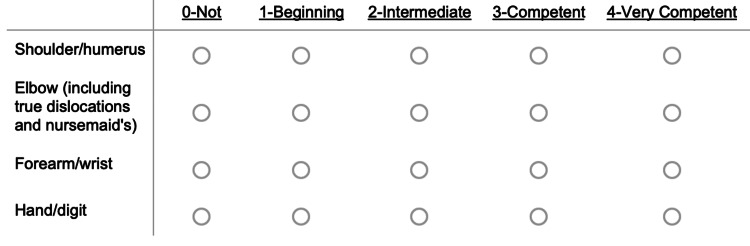
Answer Choices

**Figure 5:**
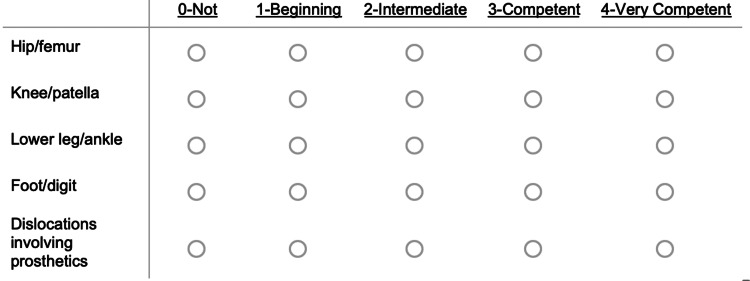
Answer Choices

## Appendices

Emergency Medicine Orthopedics Education Questionnaire

October 1, 2020

1.    Does your institution house an orthopedics residency program?

     o  YES

     o  NO

     o  No residency program, but the ortho department serves as a rotation site for residents from other programs

2.    Other than clinical rotations, which methods do you use to teach orthopedic fracture and dislocation management to your EM residents? (Select all that apply)

     ▢   Assigned reading materials in a textbook or manual

     ▢   Assigned online learning module or FOAMed materials

     ▢   Didactics: Lecture or Small Group

     ▢   Instructional Videos

     ▢   Oral cases

     ▢   Simulation on task trainers or manikins

     ▢   Simulation on high-fidelity simulators

     ▢   Procedures on live patients under supervision

     ▢   Other, Please specify _____________________________________________

3.     Do you have required orthopedics rotation required?

     o   YES > Go to 4

     o   NO > Skip to 8

4.     Which of the following services best describes your required orthopedics rotation site(s)? (Check all that apply)

     ▢   Inpatient Service

     ▢   Consult Service

     ▢   Orthopedics Clinic

     ▢   Sports Medicine Clinic

     ▢   Urgent Care 

     ▢   Emergency Department

     ▢   Pediatrics Orthopedics Service 

     ▢   Operating Room

     ▢   Other, please specify ______________________________________________

5.     Which of the following healthcare settings best describes your required orthopedics rotation site(s)? (Check all that apply)

     ▢  Academic

     ▢  Community

     ▢  County

     ▢  Military or VA

     ▢  Other, please explain __________________________________________

6.     How many weeks long is (are) your required orthopedics rotation(s)? Please answer for each level of training.

Please note: A dedicated orthopedics rotation is defined as one in which a resident's primary responsibility is to care for all musculoskeletal and orthopedic-related patient cases without any other clinical responsibilities.

7.     Does your required orthopedics rotation(s) involve dedicated orthopedic shifts? 

     o  YES

     o  NO

8.     Do you offer elective (or selective) orthopedics rotations to your residents?

     o  YES > Go to 9

     o  NO > Skip to 13

9.      Which of the following services best describes your elective orthopedics rotation site(s)? (Check all that apply)

     ▢  Inpatient Service

     ▢  Consult Service

     ▢  Orthopedics Clinic

     ▢  Sports Medicine Clinic

     ▢  Urgent Care

     ▢  Emergency Department

     ▢  Pediatrics Orthopedics Service

     ▢  Operating Room

     ▢  Support of Sports Teams or Sports Event Coverage

     ▢  Other, please specify  _____________________________________________

10.     Which of the following healthcare settings best describes your elective orthopedics rotation site(s)? (Check all that apply)

     ▢  Academic

     ▢  Community

     ▢  County

     ▢  Military or VA

     ▢  Other, please explain  ____________________________________________

11.     How many weeks long is (are) your elective orthopedics rotation(s)? Please answer for each level of training.

12.    Does your elective orthopedics rotation(s) involve dedicated orthopedic shifts? 

     o  YES

     o  NO

13.     Please describe how your residents obtain their ACGME Dislocation/Reduction numbers.

         ________________________________________________________________

14.     Has your program been accredited long enough to graduate a class of residents?

     o  YES > Go to 15

     o  NO > Skip to 22

15.    What is the average number of dislocation/reductions performed on patients you reported to the ACGME ADS for your most recent graduates (2020)?

________________________________________________________________

16.    What is the average number of dislocations/reductions performed in the laboratory you reported to the ACGME ADS for your most recent graduates (2020)?

________________________________________________________________

17.    Rating Orthopedic Competency.  Please use these Likert-type rating scales below to rate your graduating residents' competence in taking care of the following orthopedic injuries.

     Level 0=    Not competent at all

     Level 1=    Beginning competence, requires supervision 

     Level 2=    Intermediate competence, requires occasional monitoring, but not constant supervision

     Level 3=    Competent, Fairly autonomous, may need occasional check-ins 

     Level 4=    Very competent, I would allow them to treat a family member

18.    Please rate the average graduating resident from your program on their competency in performing the reduction and/or splinting procedures required for the following upper extremity joint dislocations/reductions and fracture reductions. Use the competence scale detailed above.

19.    Please rate the average graduating resident from your program on their competency in performing the reduction and/or splinting procedures required for the following lower extremity joint dislocations/reductions and fracture reductions. Use the competence scale detailed above.

20.   How do you determine your resident’s competence in managing orthopedic injuries?

________________________________________________________________

________________________________________________________________

________________________________________________________________

________________________________________________________________

________________________________________________________________

21.    Please share any additional information that you think might contribute to characterizing your orthopedics/sports medicine education. For example, if you think that your program has a unique or innovative way of teaching orthopedics, please share. 

________________________________________________________________

________________________________________________________________

________________________________________________________________

________________________________________________________________

________________________________________________________________

22.     So that we are able to credit your program as having responded and not trouble you further, please provide the name of your residency program. Once we check off your response, we will deidentify your answers. Thank you. 

________________________________________________________________

________________________________________________________________

________________________________________________________________

________________________________________________________________

________________________________________________________________

We sincerely thank you for your contribution to our project!
